# *Lactobacillus johnsonii* N6.2 and Blueberry Phytophenols Affect Lipidome and Gut Microbiota Composition of Rats Under High-Fat Diet

**DOI:** 10.3389/fnut.2021.757256

**Published:** 2021-10-14

**Authors:** Leandro Dias Teixeira, Monica F. Torrez Lamberti, Evon DeBose-Scarlett, Erol Bahadiroglu, Timothy J. Garrett, Christopher L. Gardner, Julie L. Meyer, Graciela L. Lorca, Claudio F. Gonzalez

**Affiliations:** ^1^Department of Microbiology and Cell Science, Genetics Institute, Institute of Food and Agricultural Sciences, University of Florida, Gainesville, FL, United States; ^2^Department of Pathology, Immunology, and Laboratory Medicine, College of Medicine, University of Florida, Gainesville, FL, United States; ^3^Department of Soil and Water Science, Institute of Food and Agricultural Sciences, University of Florida, Gainesville, FL, United States

**Keywords:** *Lactobacillus johnsonii* N6.2, blueberry, mediterranean diet, lipidome, microbiome

## Abstract

Obesity is considered a primary contributing factor in the development of many diseases, including cancer, diabetes, and cardiovascular illnesses. Phytochemical-rich foods, associated to healthy gastrointestinal microbiota, have been shown to reduce obesity and associated comorbidities. In the present article, we describe the effects of the probiotic *Lactobacillus johnsonii* N6.2 and blueberry extracts (BB) on the gut microbiota and lipid profile of rats under a high-fat (HF) or low-calorie (LC) diet. *L. johnsonii* was found to increase the levels of long chain fatty acids (LCFA) in the serum of all animals under HF diet, while reduced LCFA concentrations were observed in the adipose tissue of animals under HF diet supplemented with BB extracts. All animals under HF diet also showed lower protein levels of SREBP1 and SCAP when treated with *L. johnsonii*. The gut microbiota diversity, β-diversity was significantly changed by *L. johnsonii* in the presence of BB. A significant reduction in α-diversity was observed in the ileum of animals under HF diet supplemented with *L. johnsonii* and BB, while increased α-diversity was observed in the ilium of animals under LC diet supplemented with *L. johnsonii* or BB. In summary, *L. johnsonii* and BB supplementation induced significant changes in gut microbiota diversity and lipid metabolism. The phospholipids pool was the lipidome component directly affected by the interventions. The ileum and colon microbiota showed clear differences depending on the diet and the treatments examined.

## Introduction

Obesity is the leading cause of preventable death in the United States, and has linearly grown like a silent epidemy at a constant annual rate of 0.66–0.73% over the last 30 years (1). As such, it is the common origin of several chronic diseases, including cancer, diabetes, and cardiovascular illnesses. The prevalence of obesity in the population according the income levels is controversial and shows only a partial correlation with the individuals' educational degree (2–4). Undeniably, the decision to eat high energy foods is not solely dependent on social status, but instead depends on many factors (5, 6).

To establish a healthy human nutrition with scientific basis, the Dietary Guidelines for Americans (DGA) recommends switching from a western dietary pattern toward a Mediterranean dietary model. The western diet consists of highly processed foods abundant in saturated fatty acids, amended with large amount of sodium, refined sugars, food colorants, and preservatives to extend shelf life. Meanwhile the protective effect of the Mediterranean diet has been associated to the consumption of phytochemical-rich foods, including vegetables, olive oil, wine, nuts, and fruits. The health benefits of this dietary pattern have been supported by several years of research and is largely documented in the literature (7). Briefly, the extensive documentation clearly indicates that a diverse gastrointestinal microbiota associated to dietary phytophenols could play a pivotal role in promoting the desired well-balanced equilibrium (8, 9). The “signals” generated at the gastrointestinal (GI) milieu, in this favorable context, appears to be critical to achieve a positive systemic impact on health. Recent evidence suggests that several interlaced mechanisms are critical to achieve a lipid-lowering effect from the Mediterranean diet (10, 11), however, the precise pathways and mechanisms fostering the associated health benefits are yet to be fully elucidated. As such, understanding the absorption, metabolic modifications, and further circulation of lipids in the bloodstream has become an essential component to elucidate the systemic effects of these eating habits.

The composition of blood lipids could be modified by the interplay of three components: phytophenols, microbiota, and lipid catabolism in the GI system (12, 13). Obese individuals harbor a gut microbiome with reduced bacterial diversity, often described as inflammatory microbiota (14). Different studies have shown that gut dysbiosis has a central role in the pathogenesis of obesity-related metabolic alterations. This includes a direct correlation with dyslipidemia (15) and the further risk of developing chronic illnesses like type 2 diabetes and cardiovascular disease (16, 17). A gut microbiota with a large diversity is often associated with a higher quality diet (18), and the abundance of phytophenols in the Mediterranean diet, can modulate, correct, and promote diversity of commensal microorganisms in the GI tract (19). Simultaneously, the dietary plant polyphenols have demonstrated anti-obesity effects through several mechanisms. This includes suppression of adipocyte differentiation and proliferation, inhibition of fat absorption from the gut, and suppression of key enzymes that modulate lipid absorption and inhibit fatty acid oxidation in adipose tissue (20). The hosts' metabolism could be modified by altering the relationship or composition of any of these three key components, and understanding how to achieve a healthy balance among them is critical to defining rational interventions to improve lipid metabolism and correct dyslipidemia in obese individuals. Achieving a natural healthy balance may also help to minimize the side effects of lipid-lowering drugs on individuals receiving therapeutic treatments (21). Taken together, the application of this strategy has the potential to improve the health status of an increasing population of obese individuals, without abruptly changing their dietary habits.

We have previously identified and characterized important probiotic properties associated to *Lactobacillus johnsonii* N6.2 (22, 23). This strain was found to synthesize cinnamoyl esterases, improving the release and absorption of esterified bioactive phytophenols (24, 25), which healthy attributes of *L. johnsonii* N6.2 were also verified in human subjects (26). Additional evidence suggested that the benefits linked to the consumption of *L. johnsonii* N6.2 may begin with the prevention of early GI inflammatory processes. The data collected in this work demonstrate that BioBreeding Diabetes Prone (BB-DP) rats treated with *L. johnsonii* N6.2 exhibit lower levels of the active fraction of caspase-1 in ileum tissue, suggesting that *L. johnsonii* N6.2 can reduce inflammatory processes by decreasing the activation of the inflammasome (27). The inflammasome can be modulated by the presence of specific fatty acids (FAs), which have been described as one of the main regulators of metabolism, cellular signaling and immunity (28, 29). Additionally, our studies showed that rats on HF diet treated with *L. johnsonii* and phytophenols showed less metabolic syndrome biomarkers (30).

Herein, we have demonstrated that using *Lactobacillus johnsonii* N6.2 together with phytophenol-rich blueberry extract (BB) can affect the metabolism of fatty acids with systemic consequences. The effects described herein correlate with modifications of the GI microbiota composition, in response to the interventions evaluated. Furthermore, *L. johnsonii* N6.2 and blueberries directly or indirectly modulate regulatory elements in crucial regulatory pathways (like mTOR) involved to maintain overall cellular homeostasis. The results herein discussed suggest that the sterol regulatory element-binding protein 1 (SREBP-1) plays a pivotal role regulating fatty acid metabolism in the liver when the animals were fed with probiotics and phytophenols. As such, this strategy could represent an excellent and natural alternative to correct diet-caused dyslipidemia.

## Materials and Methods

### Animal Models and Housing Conditions

The animal models used in this study were Sprague-Dawley weaned pups (Envigo, East Millstone, NJ, United States). The animals were housed on ALPHA-dri bedding, two animals per cage, under the same 12 h light-dark cycles, and received the specially formulated high-fat (HF) diet or low-calorie diet (LC) and water *ad libitum*. The animal housing standards were followed as prescribed by the Association for Assessment and Accreditation of Laboratory Animal Care, and the protocols were approved by the University of Florida Institutional Animal Care and Use Committee.

### Animal Feeding Design

**Figure 1:**
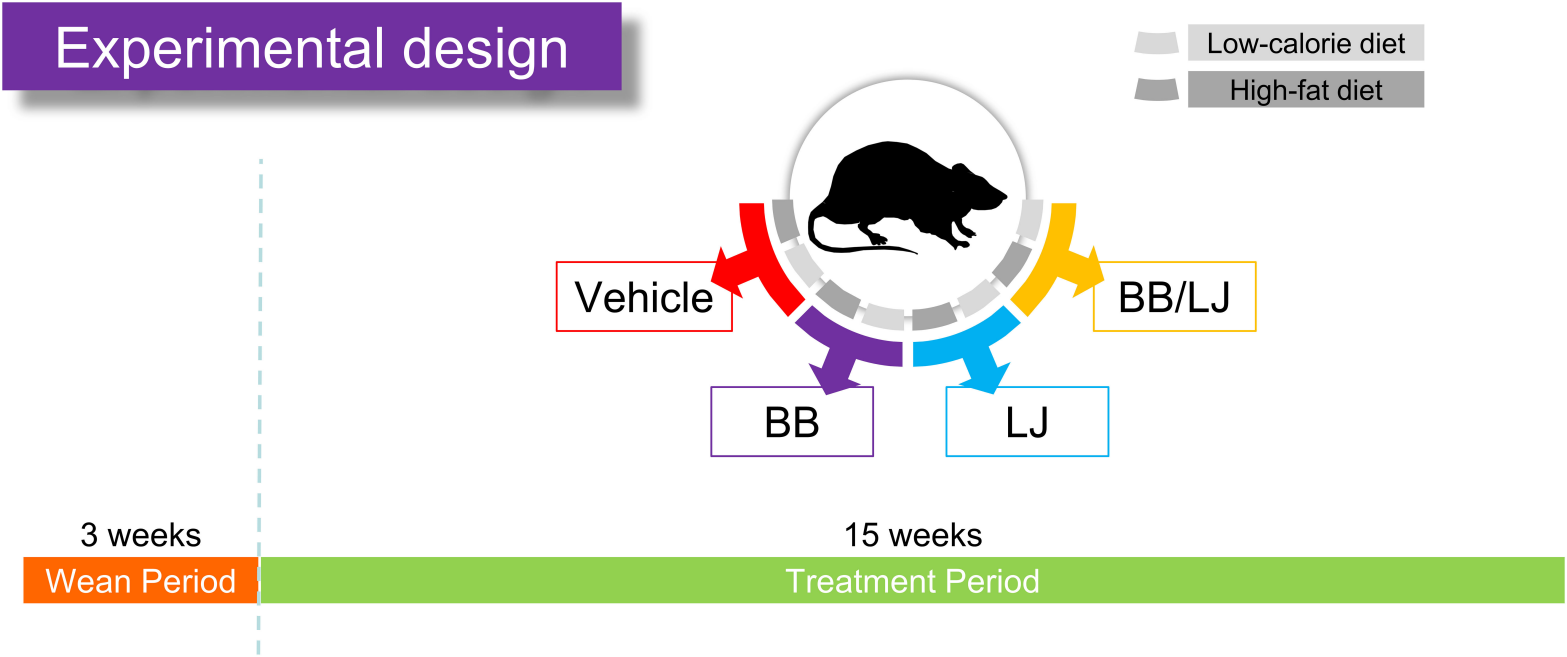
Feeding assay design. After pregnant females gave birth and pups were weaned after 3 weeks, the rats were separated into two diet groups (HF and LC diet), in which diet groups were subdivided into four treatment groups (vehicle control, BB, blueberry extract; LJ, *L. johnsonii* N6.2; BB/LJ, blueberry extract + *L. johnsonii* N6.2). Animals were fed *ad libitum* for 15 weeks and were treated three times a week.

Pregnant Sprage Dawley females were obtained from Envigo (Envigo, Denver, PA, USA). After giving birth, the mothers and their pups were housed in the same cage until weaning. Then, pups from each litter were randomly divided into eight treatment groups 21 days after birth. The animals were separated into two groups according to diet: high fat (HF) diet and low calorie (LC) diet, and four treatments (dietary interventions). Each one of the 8 groups has 14 animals. The dietary interventions were administered three times a week at the following concentrations: *L. johnsonii* N6.2 (LJ) at 10^8^ CFU/dose suspended in 100 μl PBS; blueberry extract (BB) phenols at 25 mg/kg body weight suspended in 100 μl PBS. The dose concentration was determined based on what is considered a polyphenol-rich diet, which contains around 650 mg of polyphenol per day (31). The ratio of polyphenol/kg of a rich polyphenol diet for an average human (around 62 kg) is 10.5 day or 73.5 mg/kg per week. Therefore, we administered 3 doses of 25 mg/kg in order to reach the same 73.5 mg/kg per week of a polyphenol rich diet. The interventions were conducted for 15 weeks at which point rats were sacrificed by CO_2_ inhalation followed by immediate decapitation, according the IACUC protocol. The groups were labeled as: LC (low calorie diet control), LC + blueberry extract (LC_BB), LC +L. johnsonii N6.2 (LC_LJ), LC + BB + LJ (LC_BB/LJ), HF (high fat diet control), HF + BB (HF_BB), HF + LJ (HF_LJ), and HF + BB + LJ (HF_BB/LJ) (Figure 1). LC and HF diets were formulated with the help of a nutritionist at Envigo following the Nutrient Requirements of Laboratory Animals [National Research Council (US) Subcommittee on Laboratory Animal Nutrition, 1995]. LC (TD.150312) and HF (TD.150313) (Table 1). Blood samples were collected immediately, allowed to coagulate at room temperature, and centrifuged at 4°C for 10 min at 2,000 × g. The serum samples were aliquoted and flash-frozen in liquid nitrogen. Fat tissue, as well as tissues from the gastrointestinal system, were excised and rinsed with ice-cold PBS. The tissues were flash-frozen in liquid nitrogen or preserved in RNAlater (Thermo Fisher Scientific, Waltham, MA, USA) for further mRNA analysis. All Samples were stored at −80°C until use.

**Table 1:** Macronutrient breakdown and energy densities for Low-calorie diet (LC) and high-fat diet (HF).

	**LC diet**	**HF diet**
Casein (g/kg)	170	170
Egg white solids (g/kg)	30	30
L-cystine (g/kg)	3	3
Corn starch (g/kg)	156	156
Maltodextrin (g/kg)	132	132
Sucrose (g/kg)	90	90
Soybean oil (g/kg)	40	40
Anhydrous milkfat (g/kg)	10	10
Cellulose (g/kg)	320	320
Mineral mix, AIN-93G (g/kg)	35	35
Potassium phosphate dibasic (g/kg)	1,439	1,439
Vitamin mix, AIN-93 without A, D, E (g/kg)	10	10
Choline bitartrate (g/kg)	2.5	2.5
Vitamin E (g/kg)	0.055	0.055
Vitamin D_3_ (g/kg)	0.002	0.002
Vitamin A (g/kg)	0.004	0.004
Lard (g/kg)	0	310
% Kcal from protein	26.3	13.6
%Kcal from carbohydrates	56.2	28.3
%Kcal from fat	17.5	58
*Kcal/g*	2.7	5.1

### *Lactobacillus johnsonii* N6.2 Culture

*Lactobacillus johnsonii* N6.2 was grown in MRS medium (Remel, Lenexa, KS, USA) as previously described (32). Immediately after incubation at 37°C, bacterial cells were pelleted by centrifugation, rinsed twice with PBS, and the bacterial pellet was resuspended in PBS. Aliquots were stored at −80°C for feeding assays. The CFU/mL for aliquots was determined by serial plate dilutions with PBS on MRS agar of three randomly selected aliquots. The average CFU/mL of the three aliquots was used as the concentration.

### Blueberry Extraction and Phenol Enrichment

A 50/50 (w/w) blend of freeze-dried Tifblue/Rubel blueberries was used as a phytophenol extraction source, and extractions were conducted as described by Grace et al. (33). Briefly, blueberries were blended (Waring, Inc., Torrington, CT, USA) at a ratio of 1:12 blueberry powder to acidified 70% methanol (0.5% acetic acid) w/v for 2 min. After centrifuging the mixture for 20 min at 4,000 rpm, the supernatant was transferred to a round-bottom flask. This extraction of the pellet was repeated two more times and the extracts were combined. The blueberry extract was neutralized and evaporated via Rotavapor (Buchi Rotavapor, New Castle, DE, USA), frozen at −80°C overnight, then lyophilized (Labconco, Kansas City, MO, USA). Blueberry extract powder was subsequently resuspended in PBS, aliquoted into black microcentrifuge tubes (Argos Technologies, Vernon Hills, IL, USA), and stored at −80°C until use.

### Lipid Extraction

Lipids from serum and adipose tissue of the animals were extracted according to Folch's Method, as described by Garrett et al. (34). Briefly, 100 mg of adipose tissue or 20 μL of serum samples were mixed with 2 μL of internal standard mix and transferred to a glass flask containing ice-cold methanol and ice-cold chloroform in a ratio of 1:2. After 20 min. of incubation at 4°C, water was added to the solution at a final ratio of 1:2:0.5 methanol:chloroform:water. Samples were centrifugated at 3,260 × g, at 4°C, for 10 min. The organic phase was transferred to a new glass tube, dried using nitrogen gas at 30°C, and subsequently reconstituted with 300 μl of isopropyl alcohol (IPA) and 2 μL of injection standard solution.

### Mass Spectrometry Analysis

Global lipidomic analysis was performed on a Thermo Q-Exactive Orbitrap mass spectrometer with Dionex UHPLC and autosampler. All samples were analyzed in positive and negative heated electrospray ionization with a mass resolution of 35,000 as separate injections. Separation was achieved on an Acquity BEH C18 1.7 μm, 100 x 2.1 mm column with mobile phase A as 60:40 acetonitrile:10 mM ammonium formate with 0.1% formic acid in water, and mobile phase B as 90:8:2 2-propanol:acetonitrile:10 mM ammonium formate with 0.1% formic acid in water. The flow rate was 500 μL/min with a column temperature of 50°C. Five microliter was injected for negative ions and 3 μL for positive ions.

The analysis of the data from positive and negative ion modes was done separately using LipidMatch software. First, all MS2 raw files were converted to .ms2 and MS raw files to .mzXML using MSConvert. Then, a list containing the peak intensity was generated after running mzMine on all mzXML files. An input folder that included all .ms2 files and the peak list were used to run LipidMatch to identify features. The peak areas were utilized to determine the concentration of each lipid, and the results were normalized by the sum of the peak area of each lipid.

### Western Blot Assay

Total protein extracts were prepared from tissue samples using Radio Immunoprecipitation Assay Buffer (RIPA) containing 150 mM NaCl, 50 mM Tris (pH 8), 1% Triton X-100, and 0.1% sodium dodecyl sulfate (SDS), with appropriated amounts of protease inhibitors (Sigma-Aldrich, St. Luis, MO, USA). The tissue homogenates were centrifuged at 12,000 g for 10 min at 4°C and the protein concentration measured by Pierce™ BCA Protein Assay Kit (Thermo Fisher Scientific, Waltham, USA). Ten micrograms of proteins were separated using 12.5% (v/v) sodium dodecyl sulfate-polyacrylamide gel electrophoresis (SDS-PAGE) and transferred to a polyvinylidene difluoride (PVDF) membrane (Sigma-Aldrich, St. Luis, MO, USA) using a semi dry transfer system. The membranes were blocked with 5% non-fat milk in 0.1% Tween 20, in saline Tris buffer (TBS-T) overnight at 4°C. PVDF membranes were incubated with anti-scap, anti-srebpc1, and anti-actin primary antibodies (Abcam, Cambridge, MA, USA) overnight at 4°C. TBS-T was used in all washing steps. Subsequently, the membranes were incubated with horseradish peroxidase-conjugated secondary antibody for 2 h at room temperature. The enhanced chemoluminescence method (ProSignal™ Femto; Genesee Scientific, San Diego, CA, USA) was used for visualization by both autoradiography film (Genesee Scientific, San Diego, CA, USA) and the automatic imager FluorChem R (ProteinSimple, San Jose, CA, USA). The relative luminescence intensity of the bands visualized in the membranes were quantified with ImageJ software (free Java software provided by the National Institutes of Health, Bethesda, Maryland, USA). In all cases, β-actin was used to normalize the bands of each sample.

### DNA Extraction, Library Construction, and Sequencing

DNA was extracted from stool samples collected from ileum and colon from all animals from all groups using QIAamp^®^ PowerFecal^®^ Pro DNA kit (QIAGEN), and preserved at −80°C. The following modification was applied to the extraction protocol: 100 μL of protease (20 mg/mL) from *Streptomyces griseus* (Sigma–Aldrich, Steinheim, Germany) was added to improve the lysis step (35). The mixture was incubated at 37°C for 15 min, then the samples were processed according to the kit protocol. DNA was eluted with 50 μL of water and quantified using NanoDrop One equipment (Thermo Fisher Scientific, Waltham, MA, USA). The DNA concentration was standardized to 1 ng/mL before amplification of the V4 region using primers 515F/806R barcoded for Illumina HiSeq platform (36). Library construction was made using 250 ng of each cleaned amplicon. Sequencing was performed on an Illumina MiSeq with a 300 bp paired-end protocol, using single indexing. To reduce variability and potential bias from potential sources of DNA contamination, all samples were processed with the same batch of DNA extraction kits as well as PCR reagents.

Microbiome analysis was performed using different bioinformatic tools. Briefly, Illumina adaptors were removed using Cutadapt 3.4 (37), the Silva reference database version 138 (38) was used as the reference for amplicon sequence variant (ASV) picking and for taxonomy assignment using DADA2 R package v1.10 (39). Read quality profiles were determined with DADA2, a quality score higher than 30 (Q30) was used to filter the reads, which were further trimmed 5 bases from the beginning and 10 bases from the end. Reads were merged, an ASV table was constructed, and chimeras were removed. ASVs identified as mitochondrial DNA were removed from further analyses using RStudio. Community structure was analyzed in R with phyloseq, Vegan and Microbiome packages (40, 41). Cumulative Sum Scaling was used to normalize the counts. The raw data was used as input (not normalized, not rarefied) matrix for DESeq analysis to investigate ASVs differentially abundant (42). Ordinations of PCoA and NMDS were calculated using Euclidean and Bray-Curtis dissimilarity indexes, respectively. The plots were generated with ggplot2 (43).

### Statistical Analysis

All analyses were done in RStudio 1.1.463 using 2 and 3-way interactions analysis of variance (ANOVA) to evaluate the effects and interactions of sex, diet, and treatments on the lipid profile. Significance of model terms and treatment comparisons were done with a significance level of α = 0.05, and degrees of freedom were adjusted using the Kenward–Rogers correction. Statistical power and sample size analyses were performed using G*Power 3.1.9.4. The sample size in each experiment was statistically calculated based on our previous studies with this animal model, which indicated that a minimum of 85 animals (11 animals per group) would give a power from 0.8 to 0.9 at an α level of 0.05. Based on this calculation, a total of 112 animals were randomly distributed into groups, which came out with ~14 animals per group. The microbiome study included stool samples from all 112 animals. One-way ANOVA was the first method used in order to eliminate the lipids that did not show statistical differences between the treatment groups. Secondly, 2 and 3-way ANOVA were used to evaluate the interactions between diet/sex/treatment. Finally, *post-hoc* multiple comparisons were performed with least significant differences. Metaboanalyst website was used to generate PCA and heatmap plots. GraphPad Prism 5.01 software (GraphPad Software, La Jolla, CA, USA) was used for data visualization. For the microbiome data, differences in taxonomic profiles were analyzed by Wilcoxon and Dunn tests (for two groups, mean comparison) or by Kruskal-Wallis rank sum test (for multiple groups). PERMANOVA was used to analyze clustering significance.

## Results

### The Effect of Diet and Gender on Lipid Profile in Sprague-Dawley Rats

**Figure 2:**
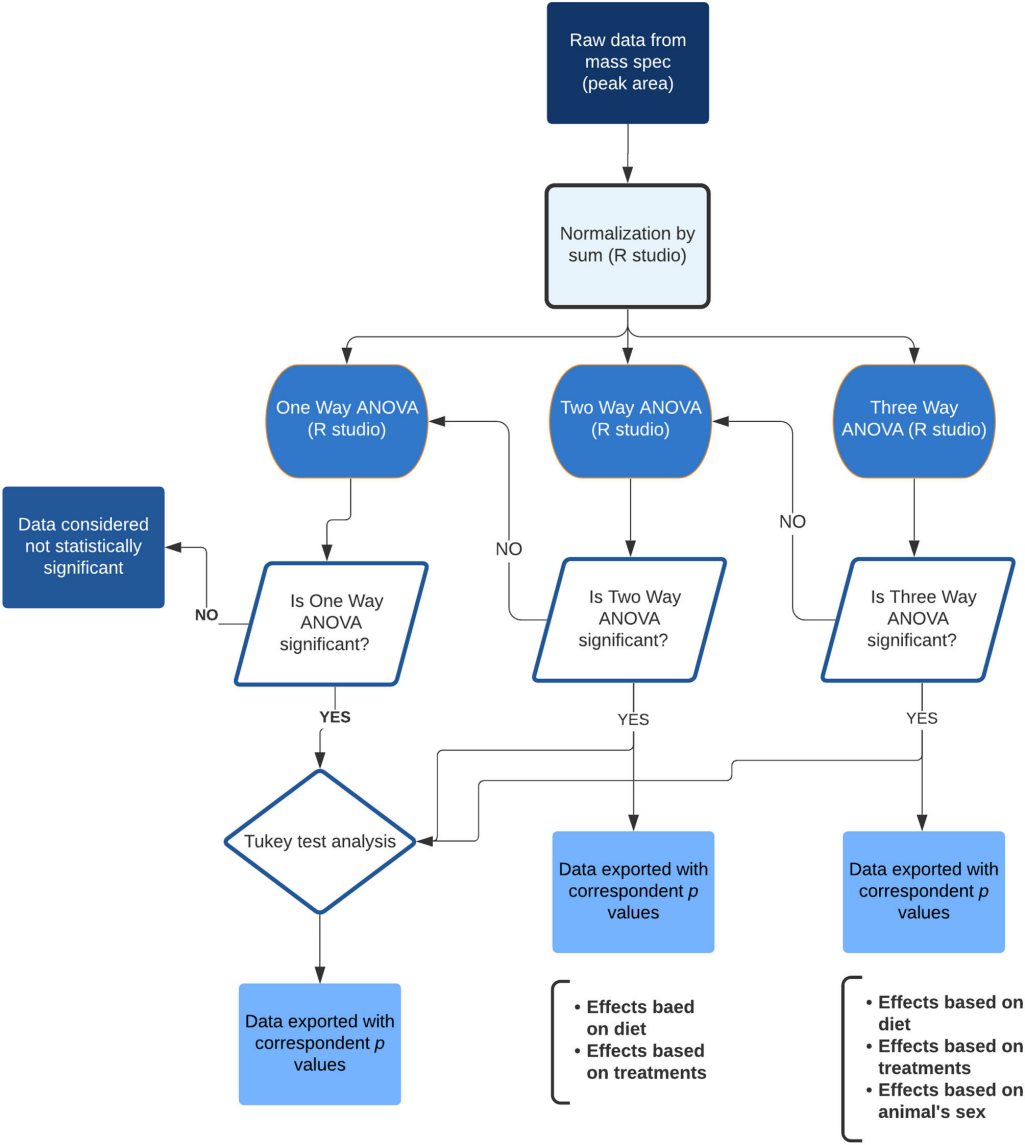
Flowchart showing the statistical analysis steps of the lipidomic data. Final data was exported to spreadsheets containing the *p-*values related to the ANOVA and effects from the diet, treatments and animal's sex, as well as the individual comparison between groups.

**Figure 3:**
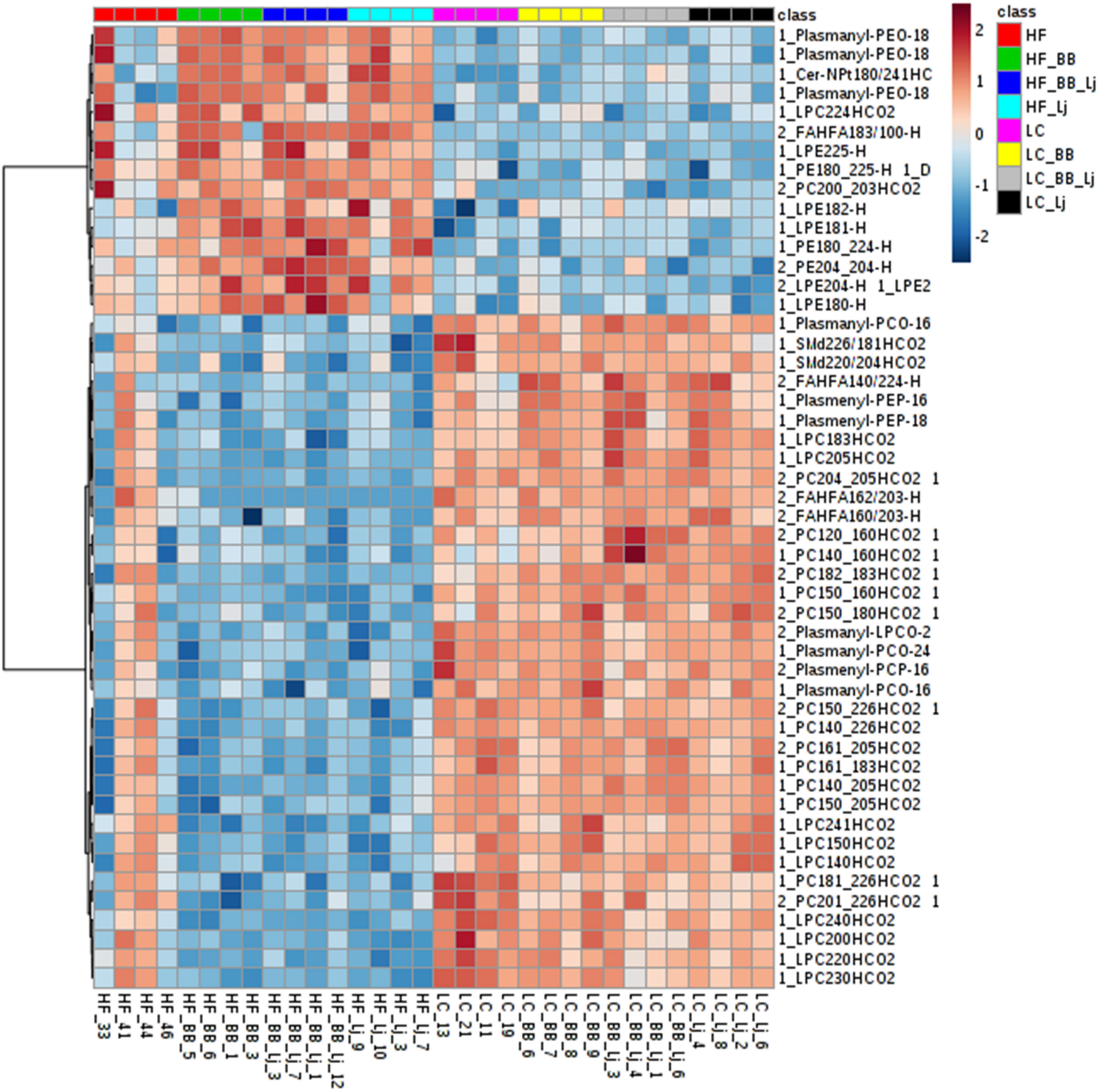
A heatmap of the top 50 lipids species observed on serum samples ranked by *T*-test to retain the most contrasting pattern. Each colored cell on the map corresponds to a concentration value. Values were measured by Euclidean distance with a Ward clustering algorithm (*n* = 6 per group). HF, high-fat diet; LC, low-calorie diet; BB, blueberry extract; LJ, *L. johnsonii* N6.2; BB/LJ, blueberry extract + *L. johnsonii* N6.2.

**Figure 4:**
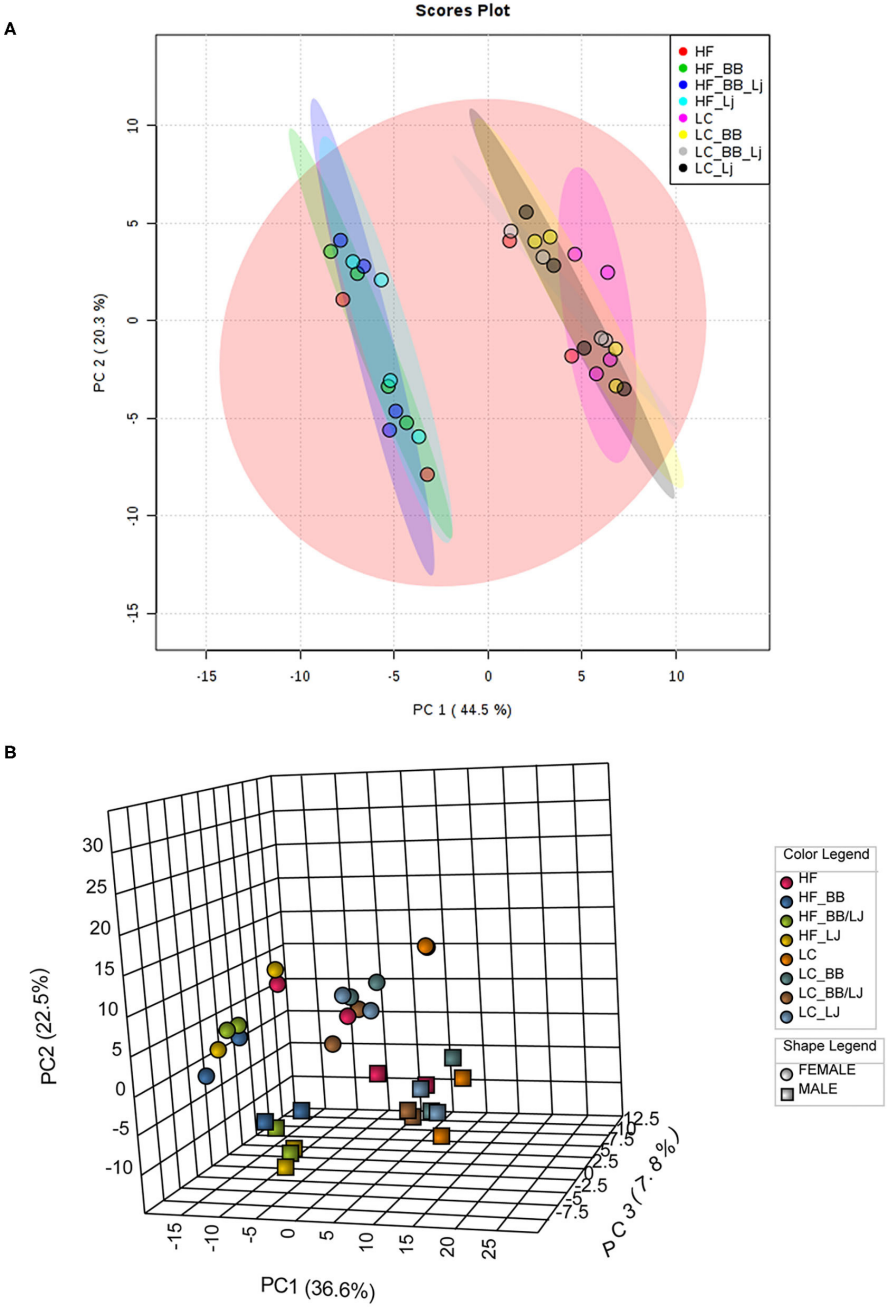
Diet affects overall lipid profile. **(A)** PCA plot; **(B)** 3D PCA plot based on sex and diet of all lipids analyzed. PC1 (44.5%) is the diet, which separates the HF from the LC diet. PC2 (20.3%) is the sex, which separates females (spheres) from the males (boxes). PC3 (19.2%) is the treatment, which separates the treatments with blueberry, *L. johnsonii* N6.2, and the combination of both. HF, high-fat diet; LC, low-calorie diet; BB, blueberry extract; LJ, *L. johnsonii* N6.2; BB/LJ, blueberry extract + *L. johnsonii* N6.2.

A total of four animals from each intervention (two diets, with four treatment groups in each diet), were randomly selected for blood serum and visceral fat lipid analysis. A total of 290 lipids from the negative scan and 510 from the positive scan were identified in serum samples, and 168 lipids from the negative scan and 605 from the positive scan were identified in the fat tissue. The lipid metabolites identified included: phospholipids (PLs), phosphatidylcholines (PCs), phosphatidylethanolamines (PEs), and their respective lysophospholipids (LPLs), lysophosphoethanolamines (LPEs), and lysophosphatydilcholines (LPCs), ceramides, and triglycerides. The lipid profile obtained was analyzed using a multivariate statistical analysis, as demonstrated in the flowchart (Figure 2). A representative heat map of the top 50 lipids (based on *T*-test score) with *p* < 0.05 from the negative ion mode is shown in Figure 3. The results indicate that diet (HF vs. LC) is the stronger component that explained the variance in the serum lipid profile of the animals. A distinctive pattern associated with the interventions individually (phytophenols/probiotic) tested in each diet was not observed. The results were validated by strong clustering obtained with a principal component analysis (PCA) (Figure 4A). A 3D PCA plot using the three significant components of the analysis revealed males and females clustering together (Figure 4B). Only two of the HF diet animals (1 male and 1 female) in the control group, clustered with the LC diet group.

**Figure 5:**
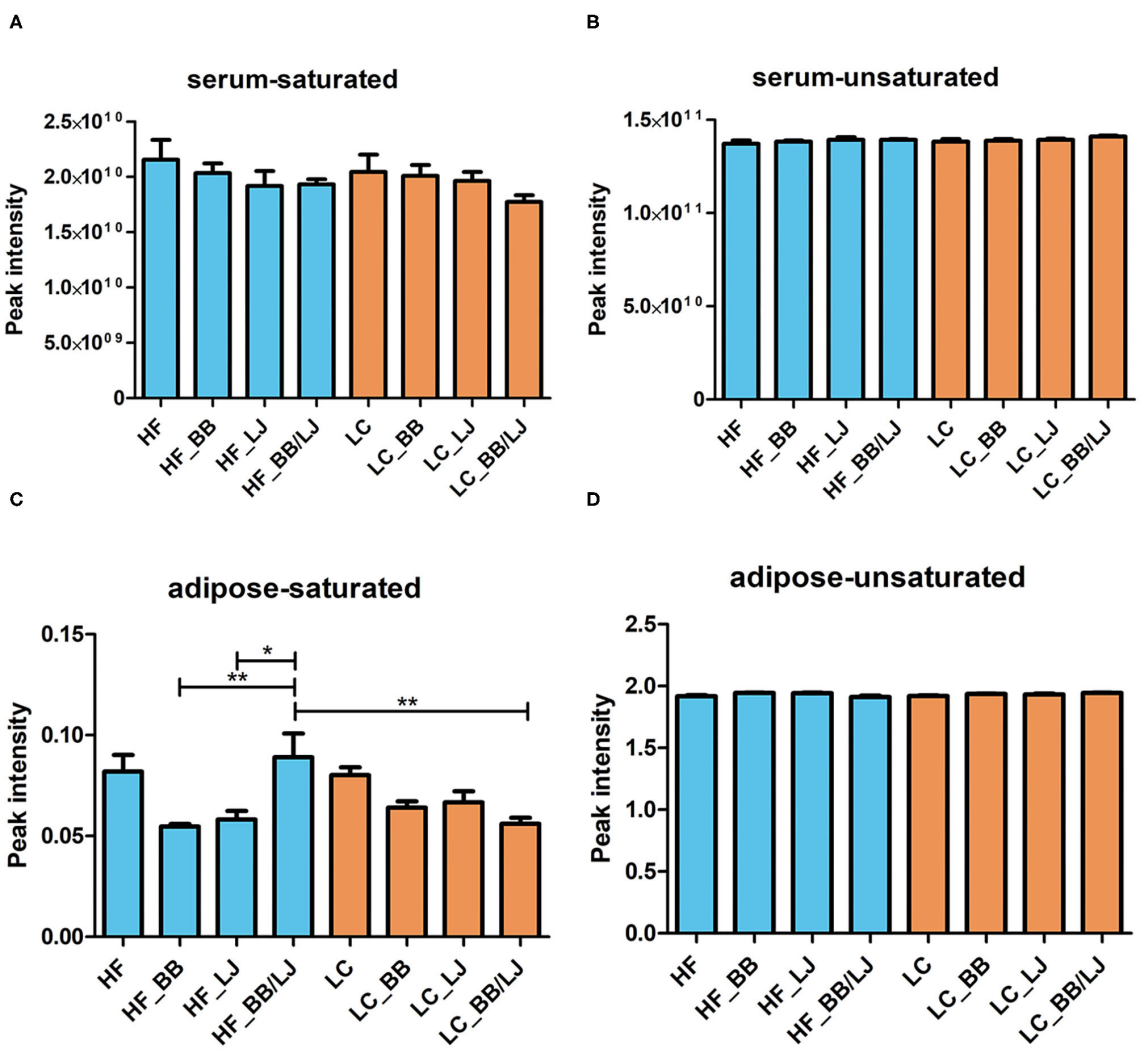
Average concentration of **(A)** saturated lipids and **(B)** unsaturated lipids on serum of the animals, as well as **(C)** saturated lipids and **(D)** unsaturated lipids on adipose tissue in the same animals. HF, high-fat diet; LC, low-calorie diet; BB, blueberry extract; LJ, *L. johnsonii* N6.2; BB/LJ, blueberry extract + *L. johnsonii* N6.2. Blue bars: animals under HF diet; orange bars: animals under LC diet. Data are summarized as mean and ±standard deviation. *p-value < 0.05; **p-value < 0.01.

Saturated and unsaturated lipids identified in the total serum lipidomic profile were used to compare the effects of the intervention in each diet. No statistically significant differences were observed in either group, however, while the unsaturated fatty acids remained practically constant in the tissues analyzed (Figure 5B), we detected variations in the saturated lipids. Saturated lipids in the blood serum decreased in all interventions, in both diets, when compared to the control groups (Figure 5A). A similar trend was observed with the saturated fats in the adipose tissue of animals fed with the LC diet, and a sharp decrease in saturated lipids was observed in the visceral adipose tissue of animals under HF diet (Figure 5C). Surprisingly, no significant difference was observed for unsaturated fats in the adipose tissue of any treatment group (Figure 5D).

### Dietary Interventions Affected Long Acyl Chain Lipid Composition in Serum and Omental Fat

**Figure 6:**
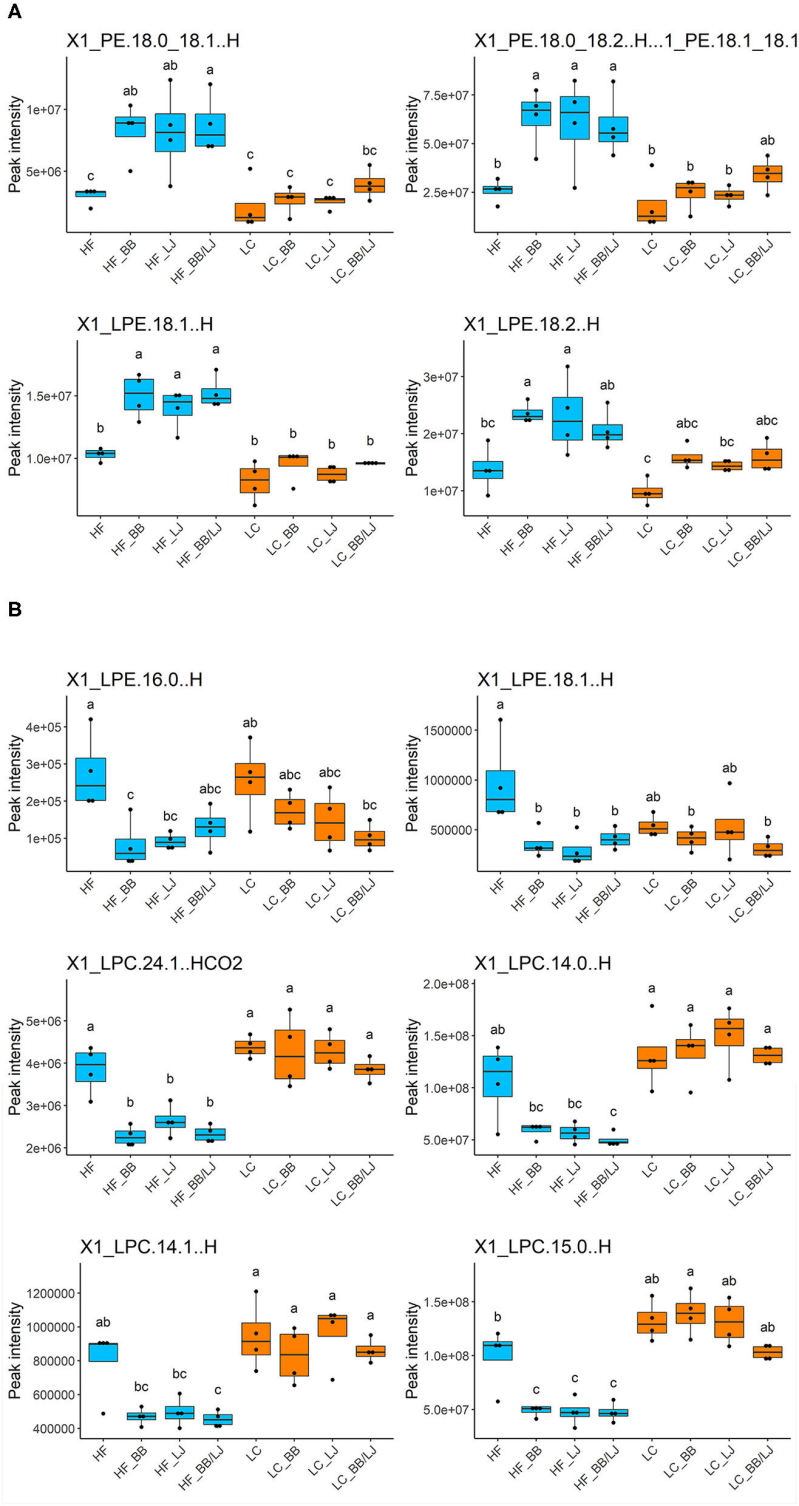
Relative concentration of phospholipids in the analyzed samples. All samples were compared based on the peak intensity of each compound identified. of **(A)** PE and LPE on serum samples and **(B)** LPE and LPC on adipose tissue. HF, high-fat diet control group; LC, low-calorie diet control group; BB, blueberry extract; LJ, *L. johnsonii* N6.2; BB/LJ, blueberry extract + L. johnsonii N6.2. Blue bars: animals under HF diet; orange bars: animals under LC diet. Data is shown as median with max/min. Different letters on top of each box plot indicate significant differences in group means from repeated-measures ANOVA.

Whole lipid analysis did not show a distinctive correlation associated to the individual interventions, however, long acyl chain (acyl chains with 13–22 carbons) (44) LPEs and PE increased in the serum samples of HF animals treated with *L. johnsonii* and BB (Figure 6A). The dual treatment (LJ+BB) demonstrated to have a clear and synergistic effect. No significant differences were observed in individuals fed with the LC diet. Interestingly, long acyl chain LPC levels in adipose tissue samples followed the opposite trend to those determined in serum samples (Supplementary Figure 1). Interestingly, treatment with *L. johnsonii* and BB, alone or combined, significantly reduced the amount of these lysophospholipids in visceral adipose tissue. A typical profile of long acyl chain LPEs and LPCs in the adipose tissue is shown in Figure 6B. We observed minimal to no significant differences for the same group of lipids in the visceral fat of animals fed under the LC diet (Figures 6A,B). These results suggest that the treatment could affect the processing and deposition of long acyl chain lipids differently, depending on diet. Therefore, to better understand the potential pathways involved in the process, the expression of key regulatory factors in liver samples were analyzed.

### *Lactobacillus johnsonii* N6.2 and Blueberries Extract Affect SREBP1 Activation

**Figure 7:**
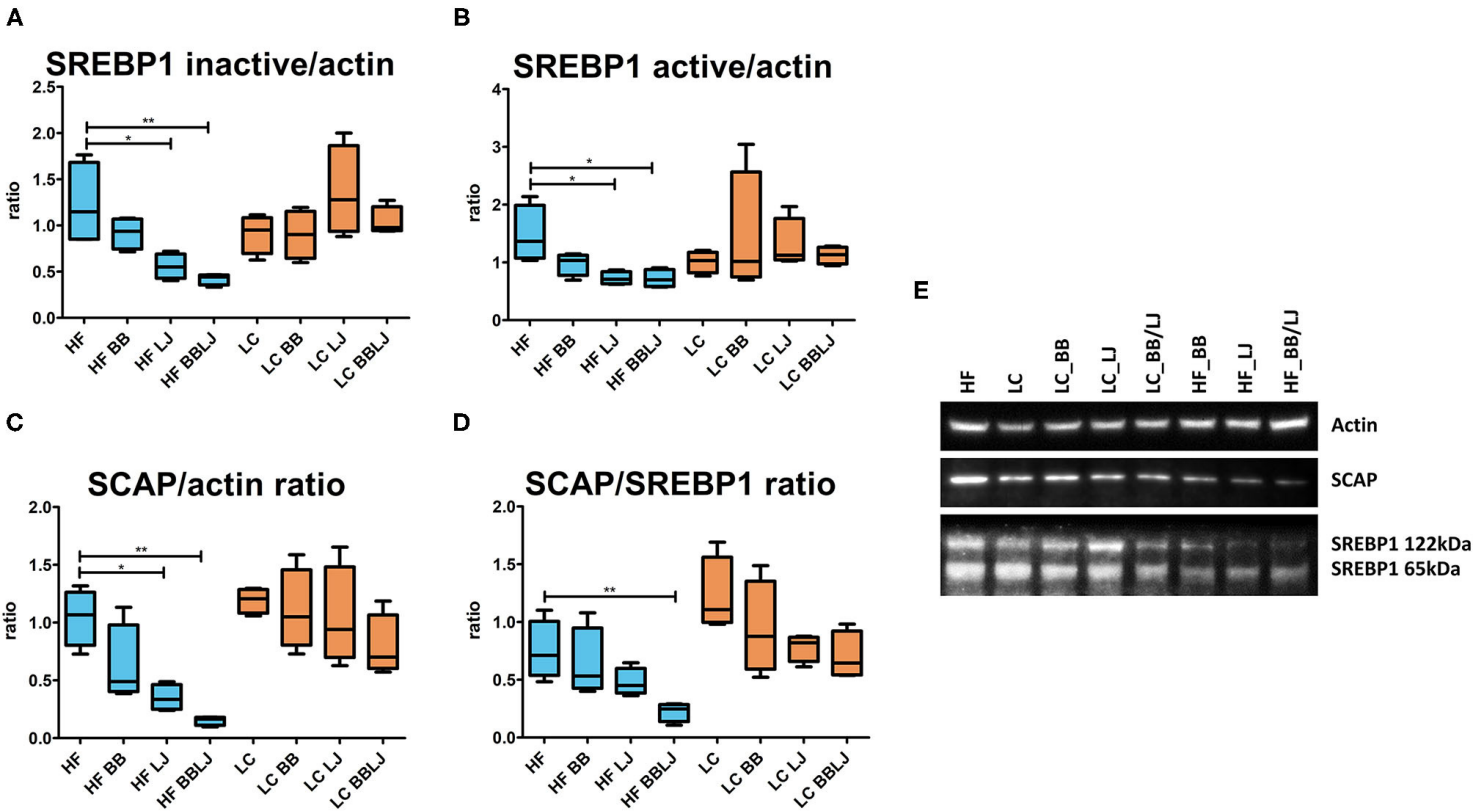
SREBP1 and SCAP protein levels determined *via* western blot. β-actin levels were used as a loading control. **(A,B)** Ratios of the inactive (122 kDa) and active (65 kDa) forms of SREBP1 over β-actin (42 kDa) band intensity, respectively. **(C)** Ratio of SCAP over actin band intensity and **(D)** over the active form of SREBP1. **(E)** Representation of the band intensity of SREBP1, SCAP, and β-actin from each treatment group. *n* = 4 from 3 independent experiments. HF, high-fat diet; LC, low-calorie diet; BB, blueberry extract; LJ, *L. johnsonii* N6.2; BB/LJ, blueberry extract + *L. johnsonii* N6.2. Blue bars: animals under HF diet; orange bars: animals under LC diet. *p-value < 0.05; **p-value < 0.01.

Lower activation of the mTOR pathway was previously observed in the GI tract of animals treated with *L. johnsonii* N6.2 and phytophenols (30). mTORC1 phosphorylation signaling is critical in the activation of SREBP1 activity in the liver and adipose tissue. The interplay of these regulatory factors connects and balances the activity of the glycolytic pathways with *de-novo* lipogenesis. The western blot analysis did not reveal significant differences in the mTOR phosphorylation cascade that modulates Akt activity in the liver tissue (Supplementary Figure 2). Although we did not detect differences in the Akt phosphorylation pattern, we observed significant differences when SREBP1 was analyzed. This membrane bound transcription factor is activated by the SREBP cleavage-activating protein (SCAP), a protease forming a complex with SREBP1 in the endoplasmic reticulum (45). Animals fed with HF diet and treated with *L. johnsonii* N6.2, alone or in combination with BB, showed lower levels of SREBP1 (HF control vs. HF_LJ: *p* < 0.05, HF control vs. HF_BB/LJ: *p* < 0.01) as well as SCAP (HF control vs. HF_LJ: *p* < 0.05, HF control vs. HF_BB/LJ: *p* < 0.01) (Figure 7). This effect was not observed in animals that were fed the LC diet *ad libitum*. Altogether, these results suggest that the treatments downregulate SREBP1 activation in liver tissue of HF diet animals via an independent mTOR mechanism. No significant differences were observed in the expression of key regulatory proteins directly involved in the modulation of lipid catabolism and biosynthesis in the same tissue. The results obtained with qRT-PCR measuring the expression of regulatory factors controlling lipids metabolism in the liver are depicted in Supplementary Figure 3.

### Ileal and Colonic Diversity Is Affected by Diet and Gender

The microbiota composition was determined in samples collected from ileum and colon contents immediately after animals were sacrificed; the V4 region of the 16S rRNA was sequenced using Illumina MiSeq technology. Samples were obtained from all animals described in Supplementary Table 1. After sequence quality control and filtering, 16,030,071 sequences were analyzed.

**Figure 8:**
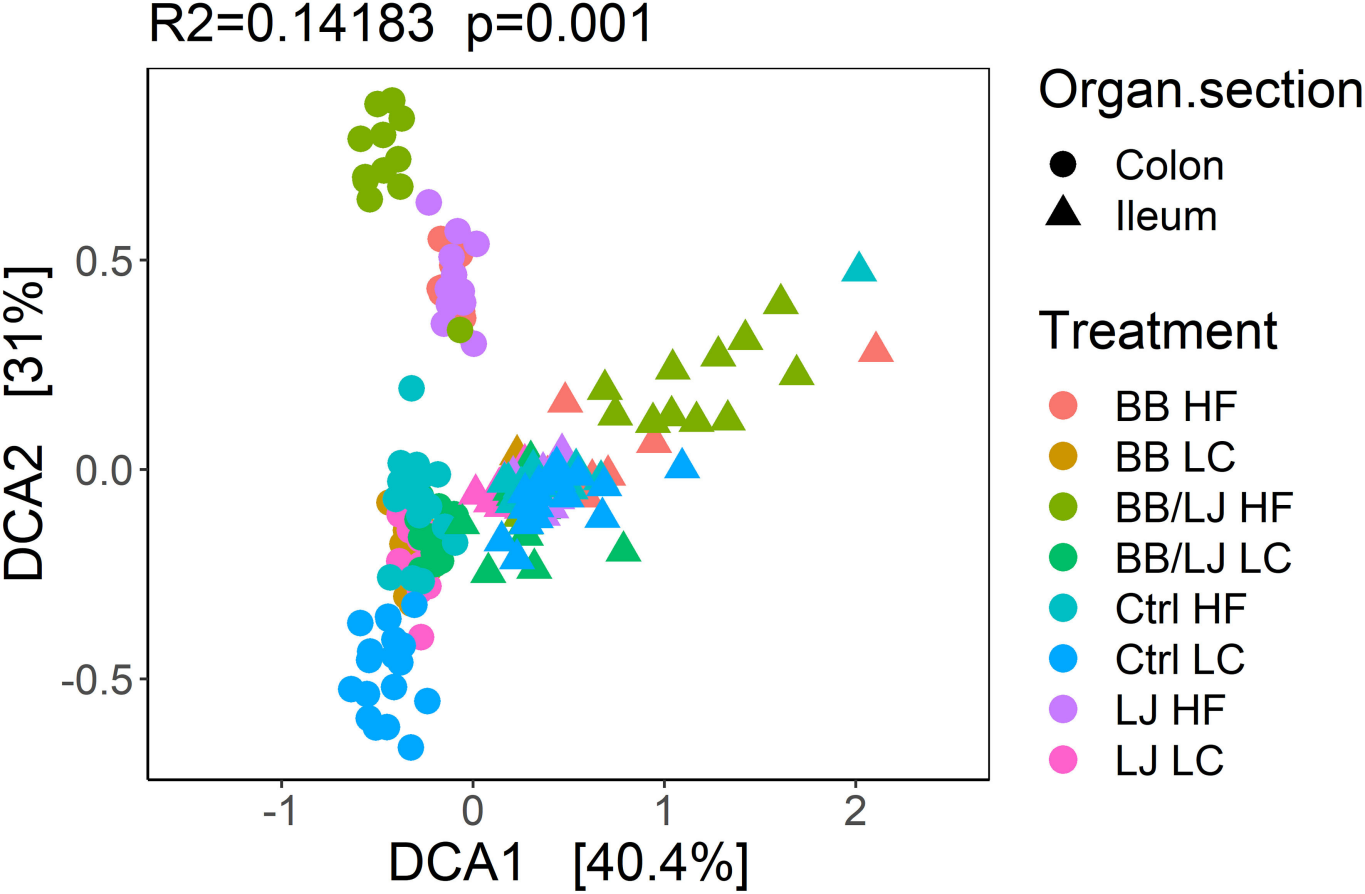
Detrended correspondence analysis (DCA) of the overall samples. PERMANOVA was used to assign statistical significance. HF, control group high-fat diet; LC, control group low-calorie diet; BB, blueberry extract; LJ, *L. johnsonii* N6.2; BB/LJ, blueberry extract + *L. johnsonii* N6.2.

A detrended correspondence analysis (DCA) was used to analyze and compare the overall structure (β-diversity) of the microbial community in the selected sections of the GI tract. The overall composition of bacterial communities present in the colon and ileum of the HF and LC treatment groups is represented in Figure 8, where clustering was observed with samples collected from the contents of the colon or ilium. While the biggest variance is explained by the location from which samples were collected within the GI tract (DCA1 40.4%), different small clusters are also observed among the colonic group of samples (Figure 8). The majority of samples collected from the ileum section of the GI tract clustered together, however, some cluster separation was observed with samples from animals in the combined BB/LJ treatment group (Figure 8).

**Figure 9:**
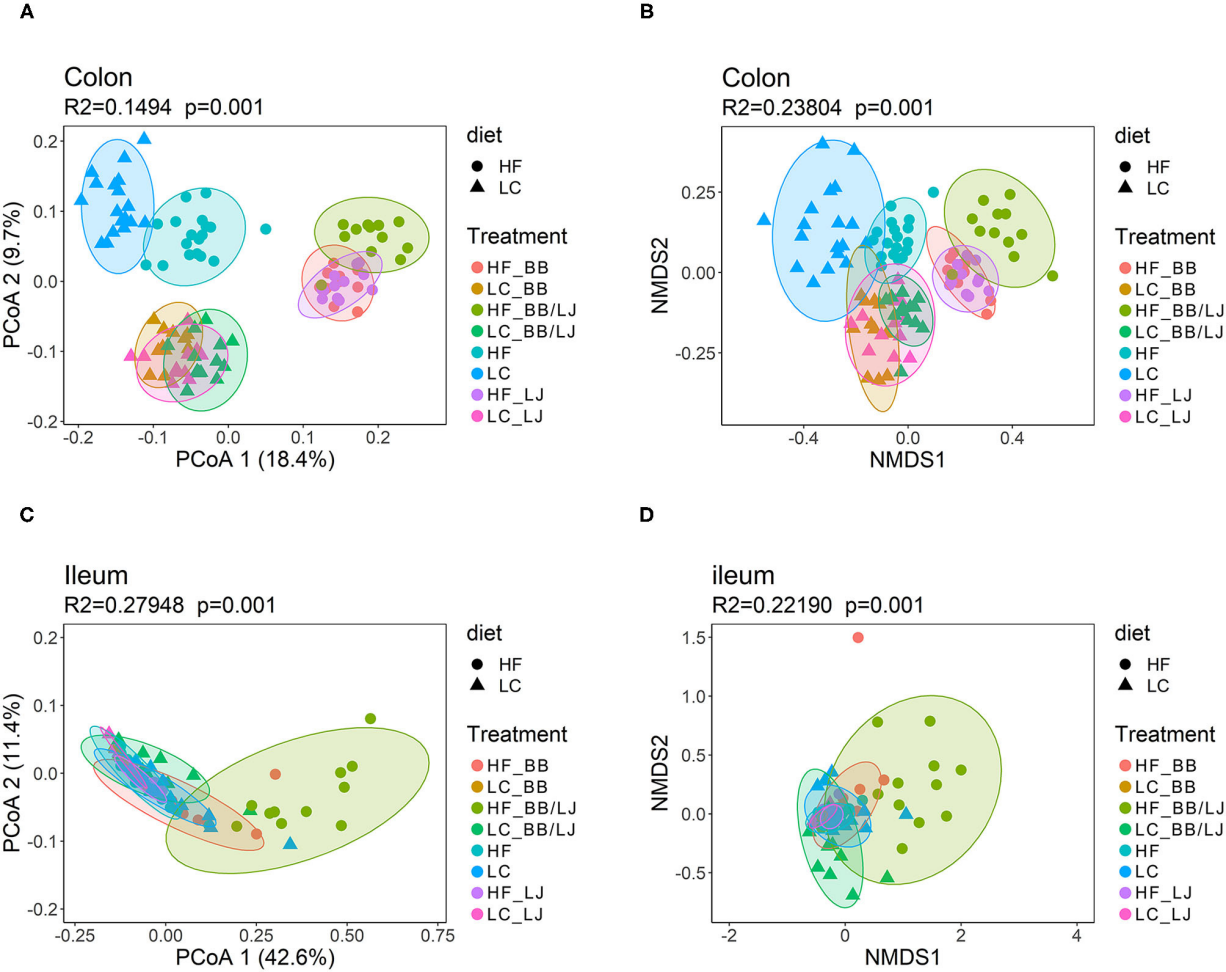
β-diversity analysis using PCoA and NMDS plots. **(A)** PCoA and **(B)** NMDS plots showing the cluster distribution of samples collected from colon. **(C)** PCoA and **(D)** NMDS plots showing the cluster distribution of samples collected from ileum. PERMANOVA was used to evaluate the statistical significance of the assay. HF = control group high-fat diet; LC = control group low-calorie diet; BB = blueberry extract; LJ = *L. johnsonii* N6.2; BB/LJ = blueberry extract + *L. johnsonii* N6.2.

The data from each sample set (colon or ileum) was subsequently analyzed separately, since the main factor driving variance was the location that samples were collected within the GI tract (Figure 9). Using a principal coordinates analysis (PCoA) and Non-metric multidimensional scaling (NMDS) we observed clear clustering corresponding to the type of treatment and diet in colon samples (Figures 9A,B). The HF and LC diets pushed the three intervention groups to form individual superclusters, where the supercluster of the LC diet was more compact than the HF cluster. Among the HF diet animals, the double intervention (BB+LJ) is clearly different from the individual use of LJ or BB. It is also noticeable that the communities of all treatments were substantially different from their respective control groups (Figures 9A,B). Interestingly, the microbial communities present in the ileum were more homogeneous overall and showed substantial changes only in the combined BB/LJ treatment group (Figures 9C,D).

The microbiota composition (α-diversity) was assessed using a non-parametric test (Kruskal-Wallis and Dunn test), where samples from male and female animals were analyzed separately based on the differences observed in the lipidomics analysis. BB induced a significant reduction in α-diversity (*p* < 0.05) in the ileum of HF diet animals (Table 2). The highest significances were obtained in ileal samples from female animals, which were found to be more sensitive to these treatments. The average index estimated for the LC diet (ileum and colon), and in the colon of HF diet animals were comparable. The Shannon index observed in the ileal region of all females from the HF diet groups were consistently lower than males under the same HF diet conditions (Table 2). Animals fed with LC diet showed an increase in the ileal α-diversity when treated with LJ or BB, when compared to the control. The Shannon index for each group of animals is summarized in Table 2.

**Table 2:** Alpha diversity of stool samples collected from colon and ileum after different treatments, expressed by Shannon Index.

	**HF diet (colon)**		**HF diet (ileum)**		**LC diet (colon)**		**LC diet (ileum)**	
	**F**	**M**	**F**	**M**	**F**	**M**	**F**	**M**
Control (Vehicle)	5.61	5.60	4.81	5.51	5.49	5.47	5.19	5.32
*L. johnsonii* (LJ)	5.52	5.66	5.37	5.43	5.01	5.55	5.52	5.58
Blueberries (BB)	5.64	5.62	4.89	5.17	5.48	5.50	5.60	5.60
*L. johnsonii* + Blueberries (BB/LJ)	5.65	5.65	3.87	4.45	5.52	5.58	5.44	5.28

*Ileum HF Females: BB vs. BB/LJ p-value = 0.02857; BB vs. LJ p-value = 0.02857; BB/LJ vs. Ctrl_HF p-value = 0.03357; BB/LJ vs. LJ p-value = 0.02857. Ileum LC Females: BB vs. Ctrl_LC p-value = 0.0003996. Ileum LC Males: BB vs. BB/LJ p-value = 0.01088; BB vs. Ctrl_LC p-value = 0.000999; BB/LJ vs. LJ p-value = 0.006993; Ctrl_LC vs. LJ p-value = 0.0001645; F, females; M, males*.

The samples obtained from each section of the GI tract were compared using a differential abundance analysis (DESeq2 analysis). We identified 155 amplicon sequence variants (ASV) differentially enriched (*p* < 0.001) between LC and HF diet animals (Supplementary Table 1). In colonic samples, 112 ASVs were differentially abundant (*p* < 0.001) between HF and LC diet animals (Supplementary Table 2). Enrichment of *Lachnospiraceae, Ocillospiraceae, Ruminoccocacea, Muribaculaceae* and *Eubacteria* families was observed in colonic samples in both diets, while increases in *Rikenellaceae, Atopobiaceae, Butyricicoccaceae, Erysipelatoclostridiaceae, Christensenellaceae, Anaerovoracaceae, Streptococcaceae, Peptostreptococcaceae, Eggerthellaceae*, and *Clostridiaceae* families were observed only in colonic samples from HF diet (Supplementary Table 2). Only three ASVs, belonging to *Ruminococcaceae* and *Lachinospiraceae* families, were differential abundant in ileal samples between HF and LC diet animals (Supplementary Table 3). The contribution of gender with each diet was analyzed individually, but no significant differences were observed.

In summary, our results indicate that ileum and colon microbiota seem to be affected in different ways by the diets and treatments examined, where ileal communities were found to be more stable and modified only by the dual treatment. A significant increase in the α-diversity was observed in the colon while decreased or minimal modifications in the Shannon index were observed in the ileum, indicating an enrichment of a more specialized microbiota.

## Discussion

Dietary components triggered important changes in the morpho-physiology of the body tissues. A direct consequence of a high-fat diet is a drastic shifting of the lipidomic biochemical profile in the omental fat and blood serum. In this study, based on the lipid profile observed as consequence of each diet, the animals grouped in two solid clusters after a double component analysis. Furthermore, they separated into four sub-clusters once the gender of each animal was incorporated in the analysis (Figure 4B). Lipids mediate several homeostatic, physiological and cellular process in the body, therefore, sexual dichotomy associated with the biological role of adipocytes is not surprising (46). Indeed, we have documented different responses in key regulatory pathways in the gastrointestinal system of animals receiving these treatments, which were linked to the animal's gender (30), however, even with the clear clustering observed, the experimental design used in this study did not provide the necessary statistical power to discuss the contribution of the dietary interventions for each animal's gender. Thus, we evaluated the nutritional value of *L. johnsonii* N6.2 and blueberry phytophenols arranged by diet (HF vs. LC).

Most of the interventions showed a decrease in the total amount of saturated fatty acids in blood serum and in fat tissue samples. This effect was observed in animals under HF and LC diets. Saturated fats are linked to several pathologies like increased risk of cardiovascular disease and cancer. Frequently, the excess of saturated fat is corrected by restrictions in the fat dietary intake, however, those dietary prescriptions usually replace the energetic value of the fats with refined carbohydrates. These changes trigger a shift in the overall metabolic state of individuals, leading to increased risk of atherogenic dyslipidemia (47). Our results indicate that dietary supplementing with phytophenols and *L. johnsonii* can help in correcting diet-induced dyslipidemia, without any additional therapies.

Analyzing the levels of each individual lipid identified in our lipidomic data showed significant changes in the phospholipid levels depending on the treatments analyzed. In general, the HF diet animals supplemented with BB and LJ together had lower levels of both LPC and LPE in adipose tissue, while significantly higher levels of PE and LPE were observed in the blood samples, suggesting that both treatments were able to change the dynamics of phospholipids in the tissues. The statistical analysis demonstrated that the supplements were significantly more efficient in animals under the HF diet. These results suggest that *L. johnsonii* and blueberry extracts induce the mobilization of lysophospholipids from adipose tissue to the plasma, helping to counterbalance the negative effects of a HF diet. Interestingly, the relative levels of some LPCs (LPC 24:1; 14:0; 14:1, and 15:0) in LC diet animals were comparable with those detected in the HF control (Figure 5B) and were not affected by the interventions. These results reinforce the idea that the phospholipid composition is differentially affected by the interventions depending on diet.

Phospholipids are common cellular components tightly regulated by the metabolic state of each individual. They have been associated with many metabolic and immunologic diseases. Fluctuations in the phospholipid profile precede the detection of autoantigens in patients with T1D (48). The levels of phospholipids were also found to be significantly lower in the cord blood of children that further developed T1D early in life. These suggest that phospholipids could be used as biomarkers in monitoring beta-cell function (49).

PE is a major constituent of cellular membranes and is a key regulator of membrane fluidity (50). It is also crucial to anchor the proteins involved in membrane fusion, and helping in the formation of the autophagosome, which is responsible for eliminating misfolded proteins and damaged organelles. PE is severely diminished in elderly patients (51) and patients with Alzheimer's disease (52). Lower levels of PE correlate with deficiencies in the formation of the autophagosome and lower capability to repair cellular components in aging (53).

Lysophospholipids are intermediate components in phospholipid metabolism and turnover, produced via phospholipase A-mediated hydrolysis of the acyl group of a phospholipid, resulting in lysophospholipid and fatty acid (54). LPE is composed of an ethanolamine head group and glycerophosphoric acid, with a saturated or unsaturated fatty acid located in the sn-1 position similar to LPC, which has a choline head group. They are minor constituents of cell membranes but have an important role in cell-mediated cell signaling and activation of other enzymes (55). LPC plays important roles in clearance of apoptotic cells, stimulating phagocyte recruitment when released during apoptosis (56). Previous studies have demonstrated low levels of LPC in serum of individuals with impaired glucose tolerance and in subjects with insulin resistant and non-alcoholic fatty liver (57, 58). Interestingly, high levels of LPC on serum have shown a negative correlation with inflammatory activities. LPC has proven to increase antioxidative capacity of high density lipoproteins (HDL), protecting low density lipoproteins from oxidation (59). Moreover, LPC seems to have a potent anti-aggregatory effect on platelets, inhibiting the release of inflammatory mediators and, consequently, reducing atherogenesis and vascular inflammation (60). Differently, high levels of LPC in tissues aggravate endothelial dysfunction and increase the expression of proinflammatory cytokines (61, 62). LPE levels have showed to be decreased in plasma of patients in chronic ischemic stroke (63), as well as in patients with T1D (64).

In summary, significant changes in lipid metabolism were induced by *L. johnsonii* N6.2 and blueberries. Furthermore, the phospholipids pool is the lipidomic component directly affected by the interventions, and these fluctuations depend on the metabolic state of the animals. The phospholipids in animals receiving the LC diet showed a different lipid profile (Figure 5B), which led us to evaluate the regulatory pathways operating in the liver.

The mTOR pathway is a key sensor regulating metabolic processes in many organs, and it plays a central role in lipid metabolism in the liver. We had reported changes in the phosphorylation pattern of several mTOR components in the GI system when animals are fed with *L. johnsonii* and phytophenol-rich blueberry extract (30). We found, in this case, that the intervention did not affect the phosphorylation pattern of mTOR components in hepatocytes (Supplementary Figure 2), and we did not detect significant differences in the gene expression of several key regulators of the fatty acid metabolism (Supplementary Figure 3). Our results showed that the lipid profile changes are regulated via a SREBP1/SCAP-dependent mechanism. The regulation of the SREBP1 appears to be independent of S6K phosphorylation, suggesting that it may be possible to modify the phospholipids inducing changes downstream of mTOR regulation. mTOR is a key pathway that regulates fundamental physiological processes, and could serve as a potential therapeutic target for the development of anti-obesity therapies (65), however, using direct mTOR inhibitors to lower lipogenesis may have adverse effects on homeostasis (66). Taken together, the use of probiotics and phenolics may represent an efficient alternative to aide in reducing the potential risk factors associated with metabolic syndrome.

The absorption of dietary lipids takes place in the upper tract of the digestive system, where bile salts emulsification and lipolytic activity is critical to enhance enterocytes lipids uptake. The changes observed in the GI microbiota may help us to explain some of the lipidomic results. In HF animals treated with LJ and BB, the drastic changes observed in beta diversity correlates with the increased amount of saturated fatty acids determined in the group. These changes in the GI microbiota suggest that the metabolism of fatty acids in the GI lumen could be significantly affected. *L. johnsonii* thrive in the proximal portion of the GI system and has the ability to synthesize a large amount of cinnamoyl esterases (24). These enzymes enhance the bioactivity of esterified phytophenols, improving its transport through cell membranes. These modifications of the GI environment may be critical to re-shape the gut microbiota composition. The significant decrease in the ileal alpha diversity, observed only in the double treatment of HF animals, suggest that some ileal species were particularly sensitive to phytophenols. Interestingly, the animals receiving a LC diet did not show significant variations in beta diversity, or in the levels of saturated/unsaturated fatty acids, suggesting the dosage of probiotic and blueberry extracts may need to be modified according to the subject's diet, to achieve the optimal results.

Diet was the determining factor in shaping the colonic β-diversity, however, the use of probiotics and blueberry extract introduced significant differences when compared to the control groups. Interestingly, in all cases the gut microbiota showed a different architecture when compared to both control groups.

Our group has established several health benefits associated to *L. johnnsonii* N.6.2 during several years. We have recorded several positive effects on animal models and successfully reproduced them in human subjects (22–27, 30, 32). Significant effects in lowering GI inflammation and boosting the immunological system were successfully demonstrated (27, 30, 67). Still, the biological molecules mediating such systemic effects has not yet been identified. The results obtained in this work suggest that such generalized effects could be linked to direct changes in the organism's lipidomic profile; however, it is yet to be determined which lipid species are working as biochemical signals that connect the changes observed in the GI milieu to the positive general impact on the subject's health.

## Data Availability Statement

The demultiplexed 16S rRNA sequencing data generated in this study are deposited in the NCBI Sequence Read Archive (SRA) under BioProject PRJNA736699. Lipidomic raw data generated in this study are deposited in the MetaboLights database under MTBLS3242.

## Ethics Statement

The animal study was reviewed and approved by University of Florida Institutional Animal Care and Use Committee.

## Author Contributions

LT, MT, ED-S, JM, and EB performed the experiments. CLG, GL, TG, and CFG contributed to the conception and design of the study. LT and MT analyzed the data. LT, MT, and CFG wrote the original draft. GL and CLG reviewed and edited the manuscript. All authors have read and agreed to the published version of the manuscript.

## Funding

This research was supported by the National Institute of Food and Agriculture, USDA (2015-67017-23182 to GL and CFG).

## Conflict of Interest

GL holds U.S. patent No. 9,474,773 on *Lactobacillus johnsonii* N6.2. The remaining authors declare that the research was conducted in the absence of any commercial or financial relationships that could be construed as a potential conflict of interest.

## Publisher's Note

All claims expressed in this article are solely those of the authors and do not necessarily represent those of their affiliated organizations, or those of the publisher, the editors and the reviewers. Any product that may be evaluated in this article, or claim that may be made by its manufacturer, is not guaranteed or endorsed by the publisher.
