# Supplementary figures and images for: Lactobacillus johnsonii N6.2 and Blueberry Phytophenols Affect Lipidome and Gut Microbiota Composition of Rats Under High-Fat Diet
Source: Front Nutr. 2021 Oct 14;8:757256. doi: 10.3389/fnut.2021.757256 (PMC8551501; doi:10.3389/fnut.2021.757256)

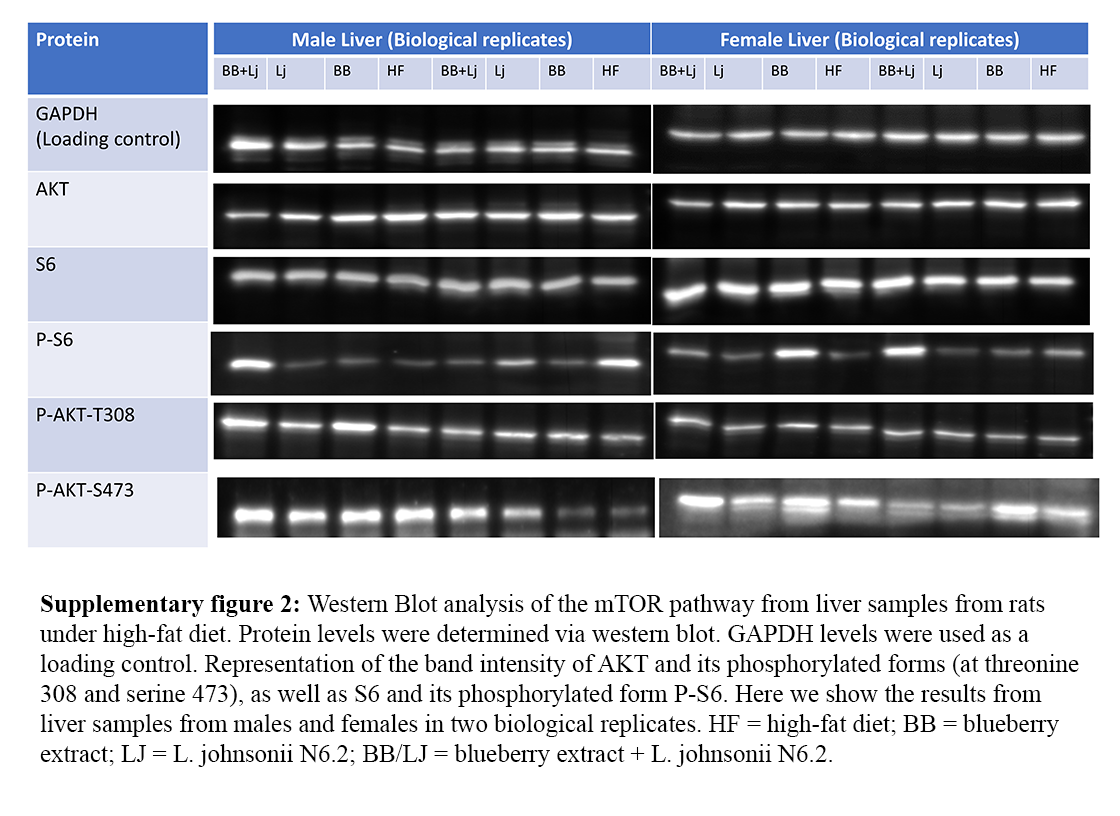

Supplement: Supplementary file 5 [file Image_2.TIF]

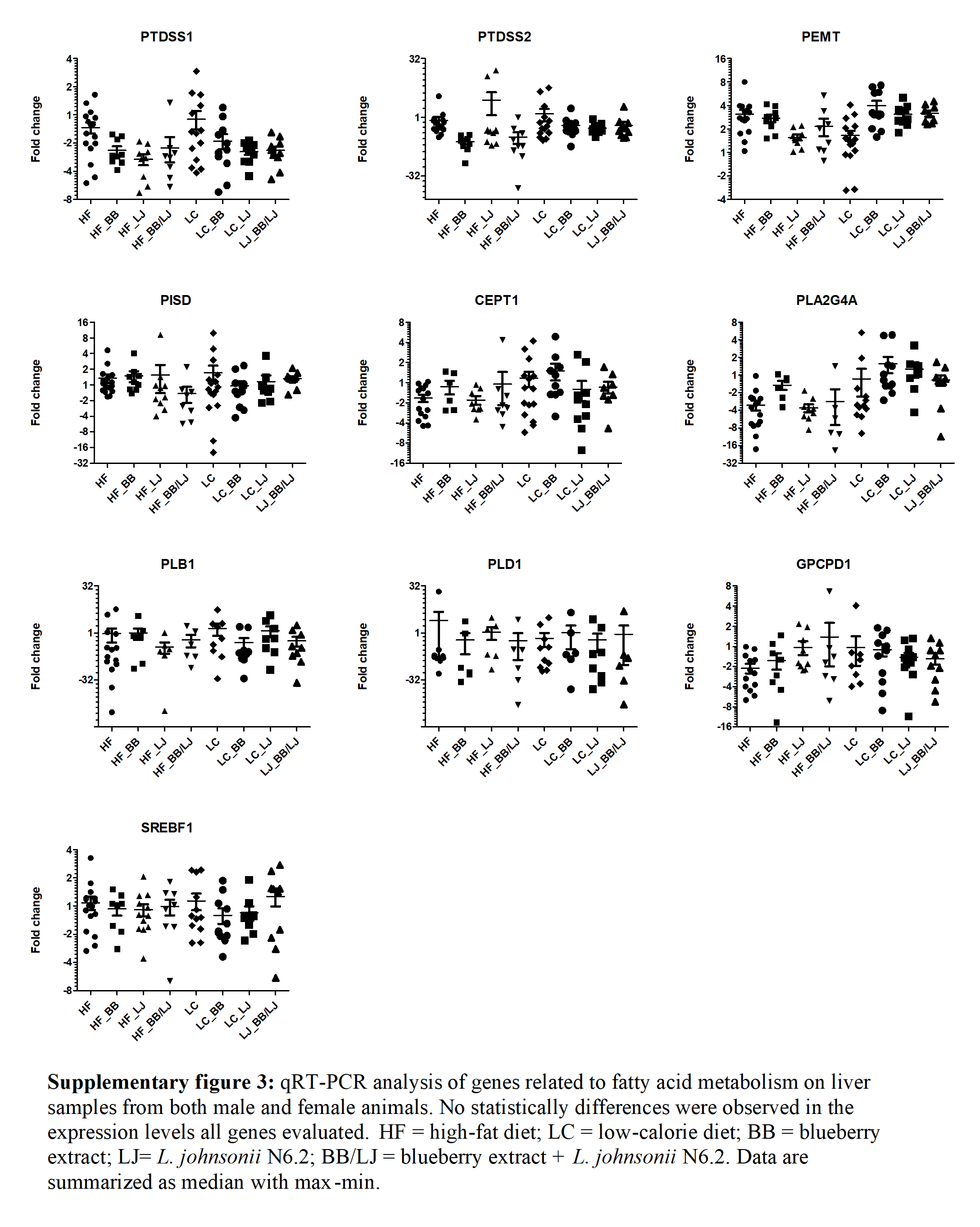

Supplement: Supplementary file 6 [file Image_3.TIF]
